# Adoption of outgroup norms provides evidence for social transmission in perinatal care practices among rural Namibian women

**DOI:** 10.1093/emph/eoaa029

**Published:** 2020-07-30

**Authors:** Renée V Hagen, Brooke A Scelza

**Affiliations:** e1 Department of Anthropology, University of California, Los Angeles, CA, USA; e2 Department of Anthropology, UCLA Center for Behavior, Evolution and Culture, Los Angeles, CA, USA

**Keywords:** cultural evolution, norm change, conformity bias, norm intervention, non-WEIRD, reproductive health

## Abstract

**Background and objectives:**

How do new ideas spread in social groups? We apply the framework of cultural evolution theory to examine what drives change in perinatal care norms among Himba women in the Kunene region of Namibia. Access to formal medical care is on the rise in this region, and medical workers regularly visit communities to promote WHO-recommended perinatal care practices. This study investigates how various forms of social transmission affect women’s uptake of medical recommendations concerning perinatal care.

**Methodology:**

Based on interviews with one hundred Himba mothers, we used Bayesian multi-level logistical regression models to examine how perceptions of group preferences, prestige ascribed to outgroup conformers, interaction with the outgroup and access to resources affect norm adoption.

**Results:**

Women who perceive medical recommendations as common in their group prefer, plan and practice these recommendations more often themselves. We observed a shift toward medical recommendations regarding birth location and contraception use that was in line with conformity bias predictions. Practices that serve as cultural identity markers persist in the population.

**Conclusions and implications:**

Norm changes, and the cultural evolutionary processes that can lead to them, are not uniform, either in process or pace. Empirical studies like this one provide important examples of how these changes reflect local culture and circumstance and are critical for better understanding the models that currently predominate in cultural evolution work. These cases can also help bridge the gap between evolutionary anthropology and public health by demonstrating where promotion and prevention campaigns might be most effective.

**Lay Summary:**

The recent promotion of WHO-recommended perinatal care practices in Namibia provides an opportunity to empirically study norm change using a cultural evolution framework. We found women adopt medical recommendations when they believe these are common in their social group. Local norms that were not discouraged persisted in the study group.

## INTRODUCTION

In its seminal 1996 report, *Care in Natural Birth: A Practical Guide*, the WHO states that the aim of care during a normal delivery is, ‘to achieve a healthy mother and child with the least possible level of intervention that is compatible with safety’ [1]. While the report notes that such care can come either inside or outside of the formal medical system, birth attendants should be properly trained, able to detect and diagnose problems and perform minor interventions. At the same time, they state uncomplicated births should take place, ‘…as close to home and her own culture as possible’, and that the laboring woman should be, ‘…accompanied by people she trusts and feels comfortable with’ [1]. In the postnatal period, while acknowledging home births and continuing to emphasize the role of kin and other support partners, there is again substantial focus on continued contact with medical professionals and promoting ‘best practices’ including exclusive breastfeeding and, when needed, treatment of mother and infant with antibiotics and antiseptics (WHO 2014). In many parts of the world, these recommendations, and the myriad policy and health interventions that stem from them, require women to straddle two normative contexts during the perinatal period: those of their home community, and those of the biomedical setting they come into contact with. Understanding how this dual context affects women’s decision-making, and their propensity to adopt new norms and practices is critical to public health. Here, we use cultural evolution theory to make predictions about what shapes women’s preferences, plans and practice related to perinatal care, set alongside predictions that are drawn from more traditional theory and practice within public health. This dual perspective allows us to parse out how an evolutionary perspective is useful for thinking about behaviors and norm change in the context of perinatal care, and where it falls short.

Cultural evolution theory has been shown to have significant explanatory power in predicting the emergence and spread of social norms [2, 3]. At the core of this theory is the idea that culture is made up of information that is learned and transmitted socially, with some cultural traits more likely to be passed on than others [2, 4]. Thus, cultural evolution theory can inform policy interventions aimed to promote behavioral change, but applied studies are rare [5, 6]. This study provides much-needed empirical data on the effect of social learning biases on norm change by examining the adoption of perinatal care norms among Himba women living in the Kunene region of northwest Namibia, where 55 out of 1000 live births do not survive beyond the first month [7]. Himba communities are currently transitioning from having very little access to formal medical care to a setting of increased access, acceptance and use.

Computer simulations and experiments have shown that learning behavior socially is often more advantageous than individual learning [8–11]. When individuals follow a social learning strategy, they do not copy cultural traits at random but are often biased toward particular types of information or individuals to learn from [12]. Social learning becomes particularly dynamic when groups interact, as this opens up the potential for new ideas to enter a population and spread [12–15]. These interactions are becoming increasingly common as urbanization and exposure to markets and communication technology increases. Public health is a rich arena to study these changes because, aside from schools, healthcare is often one of the first forums for interaction between majority and minority cultures in rural areas. Even where access to formal clinics and hospitals is limited, health workers often travel to remote areas, bringing with them recommendations, new technologies (e.g. birth control, vaccinations), and information about how to further utilize the biomedical system. As interactions with the outgroup (in this case the medical community) increase and exposure to new ideas about perinatal practice begins to penetrate a community, interesting questions emerge about how that information spreads, and whether and to what extent it supplants existing beliefs and practices. Within this arena, studying norm change in relation to perinatal care is particularly interesting from an evolutionary perspective because of its substantial impact on fitness [16–18].

### Theories of norm change

Norms and behaviors can serve as markers of group identity and facilitate interaction between group members [19–21]. Self-identification with a social group provides individuals with prescriptive norms and guidelines for appropriate behavior in a particular setting [22], and perceptions of the norms and behaviors of the members of one’s ‘ingroup’ have been shown to affect behavior in a variety of settings [23–25]. For example, Dynes *et al.* [26] found that Kenyan men and women’s use of contraceptives is influenced more by their perception of their social network’s approval of family planning, than by their own approval. For norm change to occur, group members need to shift both their perception of what the majority of others prefer and their belief about what is expected of them. There are a variety of mechanisms through which these shifts can occur, and two of the most widely discussed within evolutionary frameworks are prestige bias and conformity bias.

Prestige-biased social learning is a strategy in which individuals choose to imitate those who are known to be successful, knowledgeable or skillful [27]. Evidence for prestige-biased transmission comes from mathematical modeling and experimental studies indicating that people differentially attend to more prestigious individuals, and that cultural traits diffuse more quickly when adopted by role models or ‘opinion leaders’ [28, 29]. Interventions in public health have also shown evidence for prestige bias. Among women in rural Bangladesh, the influence of prestigious ingroup members on attitudes toward contraception has shown to be substantial: government programs to promote contraception are more successful when conducted as group discussions in the homes of local opinion leaders than when field workers visit homes individually [30]. Prestige bias could foster the adoption of a new norm in one of two ways. First, if ingroup members view outgroup members as more prestigious, outgroup norms could become pervasive through general intergroup exposure [31]. Second, in some cases, individuals’ privilege local people of high status over those from other groups [4]. In this case, if a few prestigious local actors switched to the outgroup behavior, prestige bias could occur on a local scale, with minimal general contact between the in- and outgroups [32]. This might occur if those who were highly regarded within the community also had more exposure to town or outside groups [27] (see below).

Another way in which individuals may choose which norms to adopt is by attending to the frequency of different norms in their group. For example, in studies of contraceptive uptake in Bangladesh and Poland, attitudes about contraception held by others within the social network were more predictive of women’s contraceptive use than individual-level factors [33, 34]. In another example, Rogers and Kincaid [35] show that in Korea use of particular contraceptives is clustered by village, again indicating localized copying. Conformity bias occurs when individuals display a disproportionate likelihood of copying the majority, beyond what would be expected by random copying [2, 8]. Conformity bias is predicted to be an important driver of norm change once the initial stage of norm adoption has passed and a new norm has emerged as the most common [36] and is particularly adaptive in relatively stable environments and when enough cultural models are available [11].

Where norm adoption occurs via intergroup interaction, early and frequent exposure to the outgroup can lead to increased copying of the outgroup norm. In a study on interethnic interaction in Peru, Bunce and McElreath [32] show that indigenous Matsigenka who frequently interact with an ethnic outgroup through market participation and education show more outgroup norm adoption. In their study, the timing of exposure to outgroup norms was important: earlier favorable exposure, such as through education, and positive experiences with an outgroup were associated with more outgroup adoption. Exposure to an outgroup has also been shown to influence the adoption of perinatal care practices. Existing studies on facility-based births have linked years of education with increased use of medical delivery services [37], while previous experiences of mistreatment, discrimination and inadequacies in care have been shown to decrease women’s use of services [38–40]. The effects of exposure to outgroup norms can be strengthened through interethnic assortment on norms: individuals that already hold more outgroup norms are likely to interact more with that outgroup.

The importance of social influence in promoting behavior change is also underlined in much public health research, where it is widely accepted that knowledge and information alone do not drive behavior change and that social norms can be important determinants of health behavior [41]. However, this work also focuses on practical facilitators and barriers of norm change. Individual differences in wealth and access to resources are consistently associated with whether or not women switch to practicing new healthcare practices. Bohren *et al*. [42] find that logistical and monetary barriers significantly inhibit hospital births for women in low- and middle-income societies. Women may be required to walk or use public transportation to reach medical facilities, and finding transportation during off-hours can be costly or impossible [43, 44]. Higher levels of contraceptive uptake have been found among women with more individual wealth and among women in wealthier households [45, 46, but see 47]. When deciding to spend money by visiting the hospital, not only hospital fees but also hidden costs of hospital visitation, such as medical supplies, food and lodging for family members can be prohibitive [42,43, 48]. Furthermore, the cost of hospital delivery is sometimes seen as extraneous and unnecessary when childbirth is perceived to be a non-medical event. Insufficient wealth and resource access can thus be important barriers to the practice of medically recommended behaviors among women who would prefer to adhere to them. Individuals with fewer resources may be less inclined to plan to follow more costly medical recommendations or may plan to follow a recommendation but as a result of insufficient funds be unable to.

### Norm change: preferences, plans and practices

An important link between social norm preferences and actual practices has been highlighted in the public health and development literature [49]. An individual’s preference for a social norm can be influenced by her perception of the opinions of the reference group, those people whose expectations matter to her in the situation of a norm. Important aspects of social influence are what she thinks others in the reference group do, and what she thinks others in the group approve of [49]. Situational factors can act as either barriers or facilitators of the ability to behave in accordance with preferred norms, potentially leading to discrepancy between preferences and practices. For example, discrepancy occurs when someone who prefers to give birth at home but is rushed to the hospital due to complications, or conversely, when someone intends to give birth at the hospital but is unable to secure the necessary resources to pay for transport to town.

The current study examines how social learning processes and situational factors affect the uptake of medical recommendations at three levels. We examine women’s personal preferences, their plans for their next birth and their actual practices during a previous birth. Within this context, we examine the following question: How do various cultural evolutionary processes affect preferences, plans and practices related to perinatal care among Himba mothers? We have identified a series of predictions derived from cultural evolution theory and public health to ascertain the relative influence of four key factors: conformity bias, prestige bias, interaction frequency and resource access (Table 1).

**Table 1.** Predictions and summary of results

Predictions	Preferences	Plans	Practices
Women are more likely to adopt the norm they think most others in their group support	Strong support	Strong support	Strong support
Women who would rather be like outgroup-assimilating others are more likely to adopt medical recommendations	No support	No support	No support
Women who interact with the outgroup more frequently are more likely to adopt outgroup norms	No support	No support	No support
Women who have more access to resources are more likely to adopt outgroup norms		No support	No support

## METHODOLOGY

### Study population

Around 15 000 Himba live in the Kunene region in Northwest Namibia, where most are semi-nomadic agro-pastoralists. Although Himba have a long history of interaction with neighboring ethnic groups, policies of the previous German and South African colonial governments and the post-independence Namibian government historically restricted trade and participation in the cash economy, leading to isolation from mainstream markets and commercial centers [50]. Today, Himba are becoming increasingly market-integrated, although communities vary in their distance to town, proximity to the main road and participation in the cash economy [50, 51]. Most Himba women continue to give birth in the community, and many return to their natal homes from late pregnancy until 1–6 months after birth in order to obtain help and care from their maternal kin [52, 53]. Women’s mothers commonly act as midwives, provide support in learning to breastfeed and oversee postpartum care. However, in accordance with WHO guidelines, the last decade has seen an increase in maternal healthcare programs. Professional healthcare workers visit Himba settlements in the region, where they educate women about breastfeeding and prenatal care and encourage them to give birth at the regional hospital.

The regional hospital is located in Opuwo, and a small clinic providing basic care is located in Okongwati, a village 15 km south of Omuhanga. Since 2013, a government-employed health worker has been stationed in Omuhonga, and a maternal health outreach program by Red Cross Namibia was active in the region until 2016. The hospital, clinic and nurse station both provide free contraception (injectables, pills and condoms at the clinic and hospital, condoms at the nurse station). The health worker visits compounds in Omuhonga on a monthly basis, distributes free condoms and promotes WHO recommendations. Women are encouraged to visit the hospital for pregnancy check-ups and to give birth there. Birth-assistance is provided at the clinic in emergencies only.

Data were collected between July and August 2018 in Himba settlements within a 15 km radius of the regional capital Opuwo (peri-urban sample), and in the Himba settlement of Omuhonga, which is located 122 km northwest of Opuwo (rural sample). Two sampling locations were used to be able to detect whether acculturation was higher among women living closer to town, and whether women in the peri-urban area faced fewer logistical barriers to utilizing the formal healthcare system.

### Focus groups

Two focus groups were held in Omuhonga during the first phase of the project, with a total of seven women between 18 and 42 years old. The conversations addressed norms and practices regarding behavior of pregnant women, childbirth practices and care for both mother and infant in the perinatal period. In addition, two unstructured interviews were conducted with health workers posted in Omuhonga and Opuwo. Information was acquired in these interviews about medical care available in the region, health interventions and medical outreach programs.

Together, these discussions were used to create a list of eight norm domains we believed would differ between traditional practices and local medical recommendations (Supplementary Table S1) to be used in the structured interviews. Five of the norms are commonly recommended by medical workers (giving birth in the hospital, immediate onset of breastfeeding, washing both baby and mother with water after birth and the use of contraceptives to limit fertility). Another three are traditional postnatal practices: the use of a mopane (*Colophospermum mopane*) steam for the mother to stem bleeding and prevent infection, putting a paste of cow butter and herbs on the baby’s skin (*otjizumba*), and wearing a special necklace (*oruhai*) in the weeks before birth, which is believed to protect the infant from getting their umbilical cord wrapped around their neck during birth.

### Structured interviews

An opportunistic sample of women was recruited for structured interviews, conducted in Omuhonga and the peri-urban area surrounding Opuwo. The only inclusion criterion was that participants must have given birth in the last two years. The interviews had three parts: demographic data, questions related to the key predictors and questions about perinatal preferences, plans and practices. First, we collected basic demographic information including age, education and a reproductive and marital history, and information about previous contact with health workers.

Next, we asked questions related to our four main predictor variables (Table 2). To test for the effect of perceived Himba majority preferences, we asked women which norms they think most other Himba women prefer (e.g. hospital or home birth). To measure prestige bias, we showed women two drawings: one depicting a woman in traditional Himba dress, who we explained lives in a small village, owns livestock, and has a traditional Himba lifestyle, and another showing a woman wearing western clothes, who we explained lives in Opuwo, has a salary-paid job, and is Himba but does not live a traditional Himba life and practices perinatal care norms as recommended by medical workers. We then asked participants: (i) Which woman would you prefer to be like? (ii) Which woman would you like your daughter to be like? Similar questions have been used in surveys addressing perceived prestige [54, 55]. To measure interaction with the outgroup, we recorded years of schooling, proximity to the provincial capital Opuwo, frequency of visits to Opuwo and their previous use of the hospital. Finally, individually owned wealth and access to cash informed us on women’s access to resources. Marital status was also used as a measure of resource access, as married women typically have better access to household-level wealth than unmarried women. The interview questions are further described in Table 2.

**Table 2.** Description of variables included in multivariate models and their operationalizations per prediction

Predictions	Model	Variables measured	Operationalizations
Women are more likely to adopt the norm they think most others in their group support	Conformity bias	Do you think most other Himba women prefer (traditional Himba norm) or (medical recommendation)?	Traditional Himba norm or medical recommendation (binary score 0 or 1)
Women who would rather be like outgroup-assimilating others are more likely to adopt medical recommendations	Prestige bias	Vignettes and drawings of a woman living a traditional Himba lifestyle and an outgroup-assimilating woman. Questions asked: Who would you rather be like? Who would you like your daughter to be like?	Composite score (0–2)
Women who interact with the outgroup more frequently are more likely to adopt outgroup norms	Interaction frequency	Education	Years completed (range 0–10)
		Proximity to Opuwo	Rural or peri-urban (binary 0 or 1)
		Number of visits to Opuwo over the last 6 months	Count (range 0–6)
		Hospital visitation	Never or ever visited (binary 0 or 1)
Women who interact with the outgroup more frequently are more likely to adopt outgroup norms	Resource access	Do you have N$100 right now?	No or yes (binary 0 or 1)
		If you needed money to go to the hospital, would you be able to borrow it from someone?	No or yes (binary 0 or 1)
		Tropical livestock units (TLU)	Number of cattle × 0.7 + number of sheep/goats: 0.1
		Marital status	Unmarried or married (binary 0 or 1)

**Table 3.** Demographic characteristics of the sample

*N*		
Age	Mean	26.76
	SD	7.43
	Range	16–48
Parity	Mean	3.81
	SD	2.47
	Range	1–12
Marital status	Percentage married	56
Mother alive	Percentage	90
Years of education	Mean	0.98
	SD	2.04
	Range	0–10
Tropical livestock units	Mean	1.26
	SD	2.08
	Range	0–14
Medical recommendations adopted	Preferences	6.0
	Plans	5.7
	Practices	5.3

To generate data for our three main outcome variables, we asked which perinatal care norms participants personally prefer, which norms they plan to practice for their next birth and which norms they had practiced for their most recent birth (e.g. ‘Do you think it’s better to give birth at home, or at the hospital?’, ‘For your next birth, are you planning to give birth at home or at the hospital?’, ‘For your last child, did you give birth at home or in the hospital?’).

Finally, in order to confirm that the qualitative data from our focus groups used to construct the norms questions was accurate for the focal sample, we asked respondents which norms they believed a Himba woman who follows traditional practices and what is recommended by medical workers.

### Model descriptions

We modeled the adoption of medical recommendations as a Bernoulli distribution where the probability of adopting a recommendation is a logit-linear function of the population-level effects described below. All models included varying intercepts per individual ID, per norm and per village, and population-level effects for age, age-corrected parity and a dummy variable indicating whether or not the participant’s mother was alive. This last variable was included because mothers often act as midwives and are important sources of advice, knowledge and help during the perinatal period [52, 53].

Our baseline model included only these variables. Other models included effects for our measures of conformity bias, prestige bias and access to resources as described in Table 2, with varying slopes per norm. In the conformity bias models, we compared whether women’s preferences, plans and practices matched their perceptions of the ingroup majority preference. The main predictor variable in the prestige model was a composite score of the questions who the participant wants herself and her daughter to be like (see above), and the resources model included separate variables for the different types of resources as listed above. Our models for outgroup interaction frequency included only effects for education and proximity to Opuwo, but not hospital visitation or visits to Opuwo, as visiting the hospital is required for practicing two of the medical recommendations (hospital birth and using birth control), and the hospital in Opuwo was most often utilized for this. These variables can be both a cause and an effect of adopting the medical recommendation in these cases. To avoid collider bias [56, 57], we ran a bivariate model of the effect of hospital visitation on practices excluding the norms of birth location and birth control. The same models were also run to predict women’s preferences including only those women who at their previous birth behaved conform the traditional practice, as these women had the potential to shift to the medical recommendation.

### Data analysis

Data cleaning and analysis took place in RStudio (version 1.1.463). 2% of all data points were missing, and this data were imputed with multiple imputation (R package ‘mice’). Continuous variables were centered around the mean (Supplementary Fig. S1 shows the correlation of predictors before centering). To examine the extent to which the models for conformity bias, prestige bias, outgroup interaction, resource access and the null model) explain adoption of medical recommendations in personal preferences, planned behavior and previous birth behavior, these models were tested through Bayesian logistic regression (R package ‘brms’, 58). Bivariate and multivariate models were run with 3 chains of 5000 iterations each, including a warm-up of 1000 iterations. Weak priors were assigned to all parameters to ensure model fit. We then compared the multivariate models based on their predicted out-of-sample deviance using PSIS-LOO with K-fold cross-validation, which produces robust weight scores for low sample sizes [59, 60].

After selecting the model with the lowest out-of-sample deviance, we made counterfactual predictions to calculate the absolute effects of the main predictor by varying only this predictor and holding the other variables constant. Then we subtracted its posterior predictions of women’s previous practices from the posterior predictions of their current preferences, to calculate how much the posteriors of practices and preferences differed per norm.

## RESULTS

We interviewed 101 women who had given birth within the last two years. One woman dropped out after completing <25% of the questions, leaving a sample of 100. Table 3 describes the main characteristics of our participants. Participants were between 16 and 48 years old (average 26), with parity ranging between 1 and 12 (average 3.8). Women in the rural sample owned more livestock and had completed more years of education than women in the peri-urban sample, but otherwise, the two samples were very similar (Supplementary Table S2). All women had been visited by health workers at least once before the start of our study. These visits typically included recommendations and information about hospital birth, immediate and exclusive breastfeeding and use of contraception.

Confirming our expectations, respondents showed strong agreement about which behaviors they associated with the reference categories of Himba women practicing local traditions and medical workers (Supplementary Table S1). There was one exception: respondents believed that most Himba women use at least one contraceptive method introduced by medical workers (condoms, contraceptive pill or injection), signifying adoption of these recommendations has already occurred. Out of the nine norm domains examined, on average participants had preferred, planned and practiced 6.0, 5.7 and 5.3 medical recommendations, respectively. Urban and rural women were similar in number of recommendations preferred and planned, but urban women had practiced on average one recommendation more than women in the rural sample (Supplementary Fig. S1). For all norms, women were quite accurate in reporting the preferences of the majority of other Himba women; those norms preferred by most women in our sample were also most often nominated as majority preference (Supplementary Fig. S2).

### Social transmission and norm change

**Figure 1.**
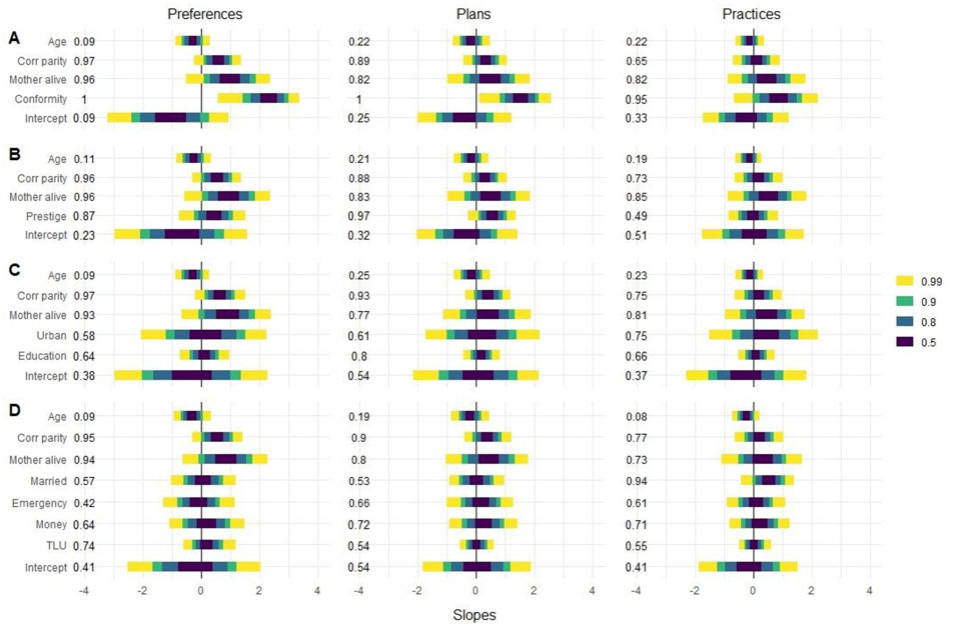
Posterior distributions of the population-level effect sizes averaged overall norms. Effect sizes for the models for (**A**) conformity bias, (**B**) prestige bias, (**C**) interaction frequency, and (**D**) access to resources are shown on a logit-scale. The colored bars indicate credible intervals 0.99 (yellow), 0.9 (green), 0.8 (blue) and 0.5 (dark blue) of the effect sizes. The probability that the effect is positive is shown on the left-hand side of the plots. See Supplementary Table S3 for the numerical values of these distributions. These effect sizes indicate the average effect on all norms and in all villages, as varying intercepts for norm and village are not included. The variation between norms and between villages is shown in Supplementary Figs S7 and S8

We fit five multivariate models (one base model and one model for each of our main predictions: conformity bias, prestige bias, outgroup interaction and access to resources; see Supplementary Fig. S3 for the correlation between predictor variables), and compared them using their predicted out-of-sample deviance. Figure 1 shows the posterior distribution of our predictors on the probability to adopt a medical recommendation on average for all norms.

Across our three outcome variables (preferences, plans and practices) the conformity bias models scored best in minimizing out-of-sample deviance and each is assigned over 99% of the model weight (Supplementary Fig. S4). These models indicate that women who believe most others have adopted more medical recommendations are more likely to prefer, plan and practice medical recommendations themselves.

We find little support for the prestige-bias hypothesis that women who would rather be like outgroup-assimilating others are more likely to adopt medical recommendations. Although this predictor seems to be positively correlated with preferences and plans to practice medical recommendations in the multivariate models, the prestige model has little weight when compared with the other models. Although the adoption of medical recommendations was slightly higher in the urban than in rural sample, the effect of living in the urban region disappears when considering other variables determining interaction frequency. In this model, years of education is weakly associated with more recommendations planned but not with preferences or practices. When we model the effect of hospital visitation and frequency of visits to Opuwo on practices excluding norms that require hospital visitation (birth location and birth control), we found no effect on practicing medical recommendations. The same results were found when only including those women who at the time of their previous birth followed the Himba tradition and who thus had the potential to shift to the medical recommendation (Supplementary Figs S5 and S6).

Because the conformity bias models were assigned over 99% of the model weight in preferences, plans and practices, we now shift our focus to these models for the rest of our analyses. Counterfactual predictions based on these models (in which the variable of interest is manipulated and the other variables are held constant) indicate that the absolute effects of women’s perception of others’ preferences are considerable (Supplementary Fig. S9). The effect is strongest for women’s own preferences, increasing her chances of preferring the same medical recommendation from 28% to 75%. It is also an important influence on the plan’s women make (41–74%), as well as on the norm they followed during their most recent birth (44–63%).

Next, we aimed to examine ambiguities between practices and preferences. In the current study, we asked women to project forward by asking them about their plans and preferences, and we also asked them about their previous behavior. This allows us to create a proxy for the baseline distribution of behaviors (frequency of past behaviors) and compare that to people’s preferences now.

**Figure 2.**
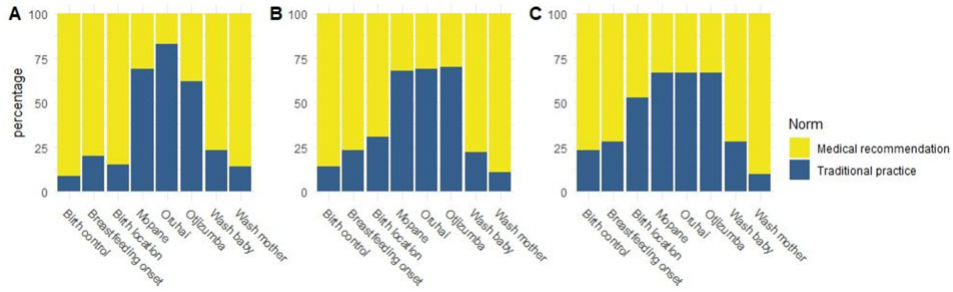
Frequency with which medical recommendations are adopted in (**A**) preferences, (**B**) plans and (**C**) practices per norm domain. Percentage of individuals who adopted the Himba tradition is displayed in blue, percentage adopting the medical recommendation is displayed in yellow

**Figure 3.**
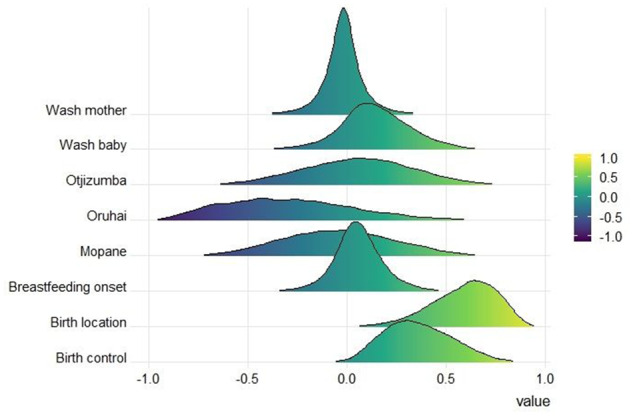
Per norm predicted difference in posteriors of practices and preferences. Positive values indicate norm domains in which the Himba tradition was more often practiced at women’s previous birth but the medical recommendation is more often preferred, and negative values indicate the medical recommendation was practiced more often before but now more women prefer the Himba tradition. Values around zero indicate no change (as many people both practiced and prefer either the Himba tradition or the medical recommendation). The parameter values used in this model are listed in Supplementary Table S4

We found considerable variation in norm adoption across the eight behaviors we studied (Fig. 2). For five of the eight norm domains, the proportions of Himba women preferring and practicing the medical recommendation do not differ significantly from each other. This means that random copying alone could produce results similar to those in our findings. However, in two cases there is strong evidence that a shift away from a traditional practice toward a preference for a medical recommendation that exceeds what would be expected from random copying (Fig. 3). When asked about use of medical contraception, 77% of women report having ever used contraceptives, whereas 91% prefer to do so now or in the future. In the second case, less than half of women (47%) reported having actually given birth in the hospital, whereas 85% of women reported a preference for giving birth in the hospital. One other norm showed clear differences between practiced and preferred behavior, but in the opposite direction: while 83% of women prefer to wear the *oruhai* necklace, only 67% did so before their last birth.

These discrepancies between current preferences and previous practices may indicate that women were not able to behave in accordance with their preferences. In the case of the *oruhai* necklace, our ethnographic data suggest that circumstantial factors can prevent women from wearing the necklace, even when they want to. For example, the family may be unable to adhere to ritual requirements necessary for the *oruhai* ceremony, or the woman may not wear the *oruhai* necklace because she is required to wear another necklace, such as the one required when you are mourning a close relative. On the other hand, the observed increase in preferences for hospital birth and the use of contraceptives in comparison to previous practices could mark a true shift in preferences as the differences are significantly larger than would be expected under random copying, although again variation in the ability to practice one’s preferred behavior or other learning biases, such as content bias may also have caused this shift.

## DISCUSSION

Choosing where to give birth, and how to feed and care for newborn infants can have important fitness consequences. Labor complications are often unpredictable and can be life-threatening, and immediate professional healthcare can greatly reduce maternal and infant mortality in the perinatal period [61]. Similarly, immediate and exclusive breastfeeding has been shown to reduce the risk of infection‐related infant mortality, because of immunological and nutritional benefits and reduced exposure to contaminated water [62]. Because of this, health practitioners are highly motivated to influence women in rural areas to take advantage of healthcare facilities and adopt WHO-recommended practices. However, empirical case studies of norm change remain rare, limiting the use of cultural evolution theory for practical purposes, a gap that we address in this study.

For norm change to occur, group members need to shift both their perception of what the majority of others prefer or do, and their belief about what is expected of them. We find strong evidence of norm change related to perinatal practices among the women in our study. The majority of women sampled had shifted many of their preferences, plans and practices to match medical recommendations.

Women are more likely to support medical recommendations when they think that most others prefer these norms, showing that social transmission is a driver of norm change. A closer examination comparing previous practices and current preferences per norm domain suggests that although some of these dynamics can also be explained as the result of random copying, we detected significant changes in some norms, particularly birth location and use of contraception. These findings are consistent with predictions from conformity-biased social learning, but we cannot exclude the possibility that other learning processes, such as content or prestige biases or random learning are what is driving the changes.

Importantly, while we have shown that preferences and practices do not always align, the cultural evolution literature currently does not address whether individuals should attend to others’ behavior or their beliefs when deciding which cultural traits to copy. As hospital births were not the most common behavior until recently, the currently stronger preference for hospital births can only result from conformity-biased learning if women based their choices on others’ preferences. It is plausible that this is the case, as the women in our study were accurate in reporting others’ preferences. Additionally, more rapid change can occur when individuals change their preferences in response to what they believe most others prefer rather than what they actually have done. This may be especially relevant to norms related to behaviors that are more spaced in time, like births. In support of this, on-the-ground ethnographic observations of this population over the last 10 years indicate that there have indeed been rapid shifts in both the uptake of contraception and utilization of the hospital for births. In a 2010 survey conducted by one of us (B.A.S.), hospital births were extremely rare even among younger women (<1% of all recorded births, *n* = 428) and contraceptive use was also quite limited. In the last 10 years access to information about both have increased dramatically.

Prestige bias, as measured in our study, did not affect the uptake of outgroup norms in our study. Forty-nine percent of women found outgroup-assimilating Himba women prestigious, but those who did so were not more likely to adopt outgroup norms themselves. These findings indicate that a bias toward outgroups or outgroup-assimilating individuals was not influencing outgroup norm adoption at the time of study, although we cannot rule out that the women in our study preferentially copied prestigious others who practice or prefer medical recommendations but otherwise are more like themselves. In addition, many of the studied outgroup norms were already quite common at the time of our study. Prestige bias may have been more important in the initial spread of new perinatal care norms, which our data do not capture. Ethnographic data suggests that this period might be quite brief. Women only began mentioning that they were being encouraged to go to the hospital to give birth in 2017, 1 year before these data were collected. In 2010, the last time systematic data on birth location was collected, only 3 women out of 118 had given birth in the hospital.

Contrary to our expectations, living closer to the provincial capital is not associated with more outgroup norm adoption. Other factors which have been shown to be important in previous studies were not predictive here. Schooling had no effect on adoption of outgroup norms. Although we had included years of education as a measure for intergroup interaction, an ethnographic perspective on the Himba context offers clues as to why education does not increase outgroup norm adoption. Most of the women who had received any education (83%) had gone to schools in villages close to Himba settlements, where they shared classrooms with students from other ethnic groups but away from urban centers.

### Disparities in norm adoption

Looking at each of our eight norm domains individually highlights some important patterns. We do not see uniformity in the adoption of new practices. While we clearly show that perinatal care norms promoted by medical workers are steadily being adopted, some traditional practices remain robust. Giving birth in the hospital, immediate initiation of breastfeeding; use of contraceptives, and washing of both mother and infant after birth had already been adopted by the majority of women in our sample at the time of this study.

Traditional practices that conflict with medical recommendations, e.g. home delivery versus hospital birth and delayed versus immediate onset of breastfeeding, are most susceptible to replacement. But as our results show, perceptions of a majority preference for the new norm do not invariably lead to the erosion of traditional practice. Traditional norms that are not in conflict with new norms, such as wearing a traditional *oruhai* necklace in the perinatal period, and using a mixture of herbs to clean the baby, persist among Himba women. These norms are not discouraged by outgroups, are relatively cost-free and are important cultural markers signaling Himba identity. Whether a traditional norm stays or goes likely depends on the perceived benefits of change, as well as the costs of maintaining the tradition.

The gap observed between previous practices and current preferences regarding birth control, birth location and use of the *oruhai* necklace is possible evidence of conformity bias; however, another explanation is that there were unmeasured barriers preventing women from enacting their preferences. Some women cited situational barriers as reasons why they were unable to use the hospital, such as not being able to get to the hospital in time. Given this, we expected that access to resources might have an effect on outgroup norm adoption [42], but we did not find evidence for this. It may be that the measures of resource access we used were too crude, or that logistical barriers such as access to transportation, or circumstantial factors (e.g. going into labor earlier than expected) could be leading to the difference between plans and practice. Similarly, in a highly mobile, rural population like this, women may find that uptake of contraception is difficult even when desired. Circumstantial barriers also likely played a role in the difference between preferences and practices in wearing the *oruhai* necklace, a norm domain in which the traditional practice remains widespread. These examples highlight the importance of bringing ethnographic context into empirical studies of cultural evolution.

### Study limitations

In this study, we examined which social learning biases influence the spread of a set of norms that were already fairly common in the population. We did not address how new norms were first adopted into the social group and our study may have missed important processes that were prominent during the initial spread of new norms. In order for a new norm to first enter a group, some individuals need to be ‘innovators’. When unfamiliar norms become available through contact with outgroups, several mechanisms can lead to the introduction of this norm by group members. When social learning is biased by perceptions of prestige, individuals may copy norms of outgroups that are perceived as prestigious, or they may ascribe prestige to and copy those members of their own group who themselves display outgroup norms. Alternatively, content or payoff biases can instigate the introduction of an outgroup norm. Conformity bias can only act on norms that are already common in an individual’s reference group [36], although when some group members include outgroup individuals in their reference group, conformity bias could lead to the adoption of outgroup norms into a social group.

Second, we should consider the possibility that an unmeasured confounder affected both the respondent’s belief and the beliefs of those whose preferences she seems to copy. All women in our sample have received health recommendations through government-led and NGO outreach programs, but this study did not capture fine-grained information on individual variation in the frequency, intensity or content of contact with health workers. However, our model indicated an effect of conformity bias that is beyond the effect of living in the same village. Therefore, although we cannot rule out that similarity in experiences between women and their peers not addressed in this study led to the consistency in their beliefs, we believe this effect is minimal.

Third, our measures of prestige and conformity bias, as in many empirical studies, are imperfect, as we aimed to balance ecological validity and ease of interpretation with theoretical precision. As Acerbi *et al*. [63] note, patterns in norm change that are expected under conformity bias may also be created by other processes, such as individual preferences for one variant or selective copying of a subset of the population, for example as a result of prestige bias. We have aimed to rule out prestige bias as a significant driver of norm change, but subsequent research is still needed to provide more definite proof of conformity bias. Our measure for prestige was based on questions asking about a participant’s wish for herself or her daughter to be like someone who practices medical recommendations. While these questions have been part of other surveys addressing notions of prestige, these studies also included interview items that address respect and admiration [54, 55]. It is possible that because our measure was more limited, we missed other important aspects of this concept.

## CONCLUSIONS AND IMPLICATIONS

Most previous applications of cultural evolution theory related to reproduction have focused on contraceptive uptake [34, 64, 65]. Our study shows that this theoretical framework can be usefully applied to consider other kinds of perinatal care norms and practices. Access to healthcare is on the rise for Himba women in Northwest Namibia, and women are frequently approached by governmental and non-governmental health workers promoting WHO-recommended perinatal care practices, such as hospital birth and immediate and 6 months of exclusive breastfeeding. We find that conformity bias may be one important factor in the adoption of WHO-recommended perinatal care norms among Himba women. However, the promotion of new behavior does not necessarily lead to the erosion of all traditional perinatal care practices: women in our study continued to practice traditional norms that were not in conflict with WHO-recommended norms. Norm change interventions can make use of these findings by increasing awareness of the attitudes and behavior of individuals in the target population once the majority has adopted the recommended behavior, as this may encourage nonadopters to adhere to the new norms of their peers.

Cultural evolution theory has the potential to inform public policy aimed to instigate behavior change [5, 6]. Our research suggests that once a new norm is common in the group, initially hesitant individuals will soon follow. By looking not only at behavior but also at people’s plans and preferences we also highlight differences in these three outcomes, which may be useful in future models. We suggest that in some cases people may be attending more to changes in majority preferences, rather than actions, which could lead to rapid norm change. This may be particularly relevant in cases where behaviors are rare (as with births). Subsequent research could parse these differences more clearly by using longitudinal data on both preferences and behavior.

## Supplementary data

Supplementary data is available at *EMPH* online.

## Supplementary Material

eoaa029_Supplementary_Data
