# Supplementary material for: Adoption of outgroup norms provides evidence for social transmission in perinatal care practices among rural Namibian women
Source: Evol Med Public Health. 2020 Jul 30;2020(1):161–73. doi: 10.1093/emph/eoaa029 (PMC7547623; doi:10.1093/emph/eoaa029)
Supplement: eoaa029_Supplementary_Data [file eoaa029_supplementary_data.docx]

# SUPPLEMENTARY MATERIALS

Data and code can be found at <https://osf.io/y85bq/>.

## Agreement on Himba traditions and medical recommendations

Table S1. Overview of Himba traditions and medical recommendations addressed. Numbers in parentheses indicate the percentage of women who believe Himba women living a traditional lifestyle practice and medical workers recommend a norm for each domain. Women’s perceptions of traditional practices and medical recommendations aligned with our expectations, except for one domain. Most women believe that the use of contraceptives is common among those living a more traditional lifestyle. In line with our expectations, most women thought that medical workers do not promote or try to dissuade women from wearing the oruhai necklace.

| Norm domain | Explanation | Traditional Himba practice | Medical recommendation |
| --- | --- | --- | --- |
| Birth location | Home or hospital birth. | Home  (84) | Hospital  (95) |
| Breastfeeding onset | Breastfeeding is started on the day of the birth (immediate) or at least one day after the birth (delayed). | Delayed  (65) | Immediate  (75) |
| Contraceptive uptake | Use of condoms or anti-conception pill or injection. | Not practiced  (35) | Practiced  (96) |
| Mopane steam bath | After giving birth, women take a steam bath by squatting over a hole in the ground that is filled with hot stones and soaked leaves of mopane (*Colophospermum mopane*) to create steam. | Practiced  (90) | Not practiced  (89) |
| Oruhai necklace | Women replace their regular necklaces with a looser oruhai necklace in the weeks before the birth. The emic explanation of this behavior is that it prevents the umbilical cord from tying around the infant’s neck during birth. | Practiced  (97) | No opinion  (56) |
| Otjizumba | A paste of cow butter and herbs is rubbed on the infant’s skin. | Practiced  (89) | Not practiced  (87) |
| Washing mother | The mother washes herself with water after giving birth. | Not practiced (62) | Practiced  (94) |
| Washing infant | The infant is washed with water after the birth. | Not practiced  (65) | Practiced  (91) |

## Study sample

Table S2. Demographic characteristics of the sample divided between rural and peri-urban participants.

|  |  | Rural sample | Peri-urban sample |
| --- | --- | --- | --- |
| N |  | 44 | 56 |
| Age | Mean | 25.98 | 26.82 |
|  | SD | 7.35 | 7.35 |
|  | Range | 16-47 | 16-48 |
| Parity | Mean | 3.8 | 3.8 |
|  | SD | 2.32 | 2.57 |
|  | Range | 1-9 | 1-12 |
| Marital status | Percentage married | 55% | 57% |
| Mother alive | Percentage | 91% | 91% |
| Years of education | Mean | 1.43 | 0.62 |
|  | SD | 2.07 | 1.97 |
|  | Range | 0-7 | 0-10 |
| Tropical livestock units | Mean | 2.03 | 0.69 |
|  | SD | 2.57 | 1.13 |
|  | Range | 0-14 | 0-6 |

## Norm frequencies


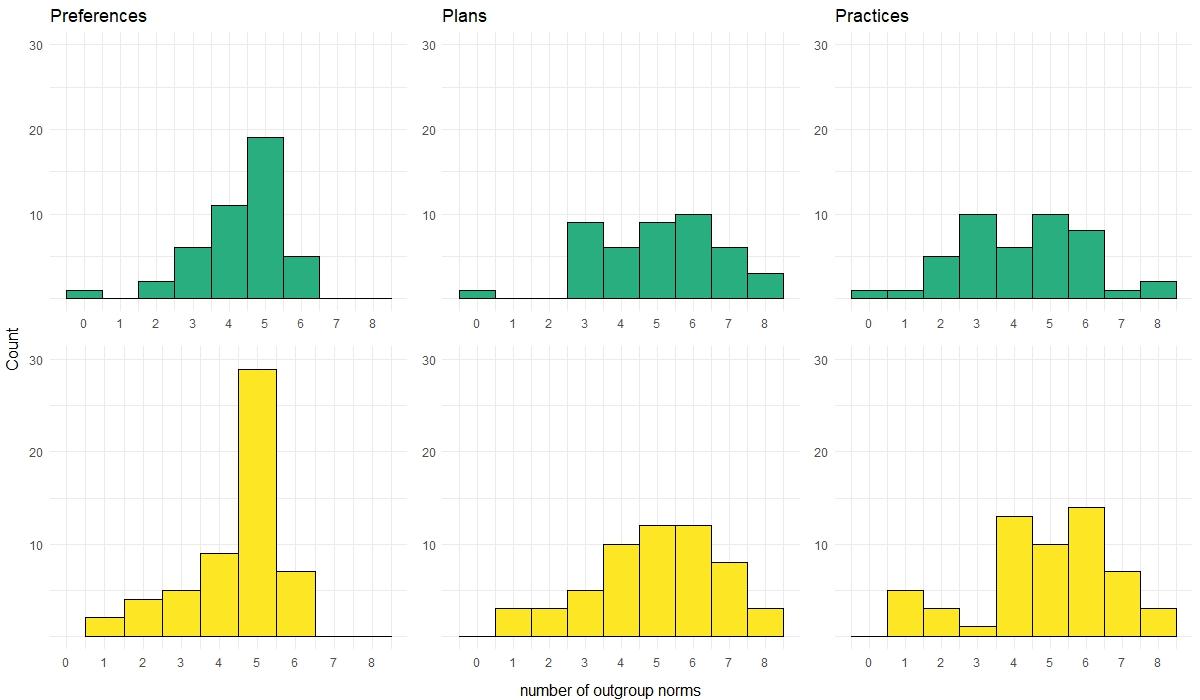


Figure S1. Histograms of number of medical recommendations preferred, planned and practiced in the rural (green) and peri-urban (yellow) sample.

## Perceived majority preference and self-reported preferences


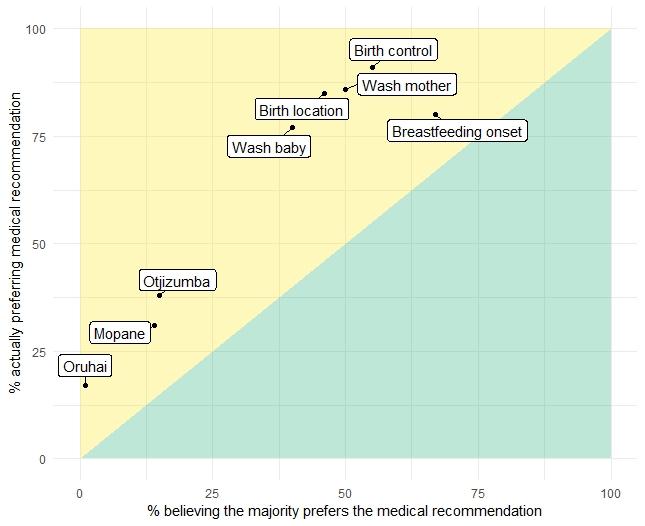


Figure S2. A per-norm comparison of women’s perceptions of medical recommendations preferred by the majority of Himba women, and women’s own self-reported preferences. All norms fall in the yellow area, indicating that the norms that more participants self-report to prefer are also more often thought to be the majority-preferred norm.

## Multivariate models


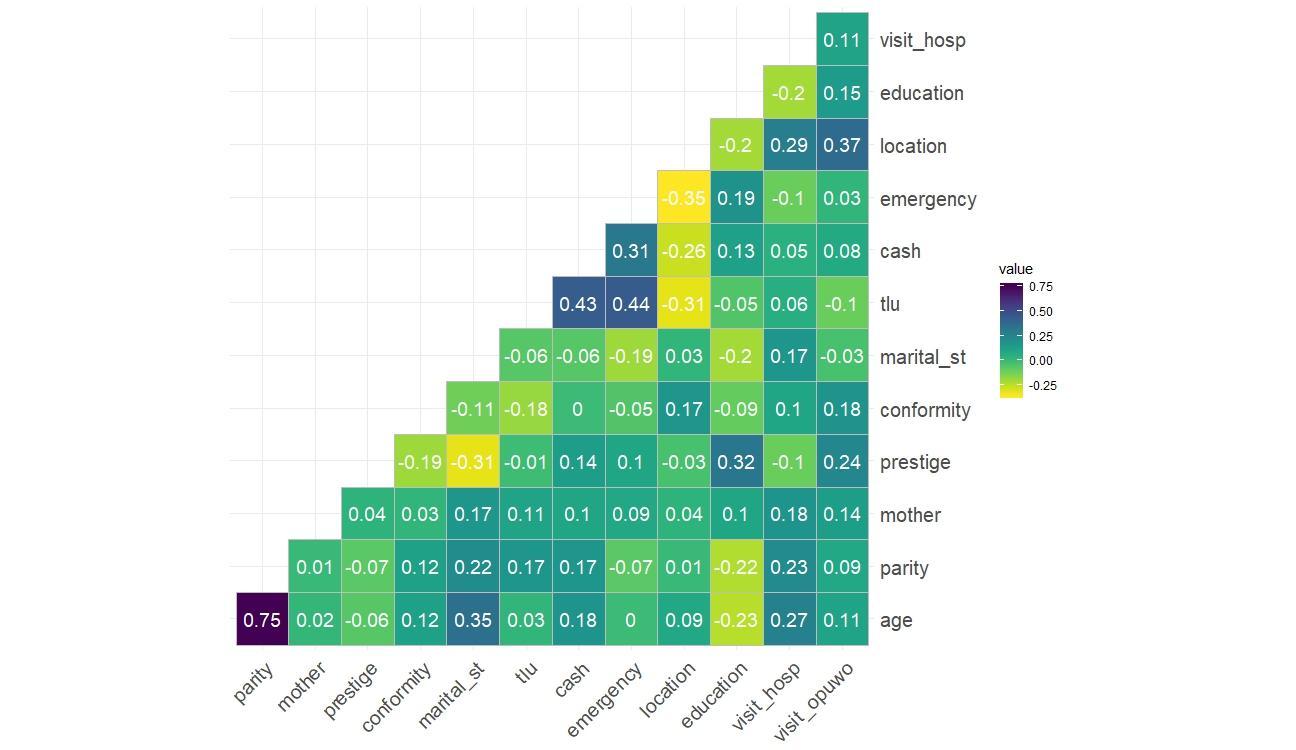


Figure S3. Correlation plot of the predictor variables used in the multivariate models before scaling.

| **A** | 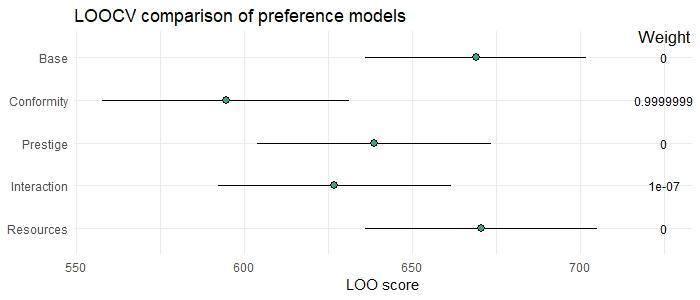 |
| --- | --- |
| **B** | 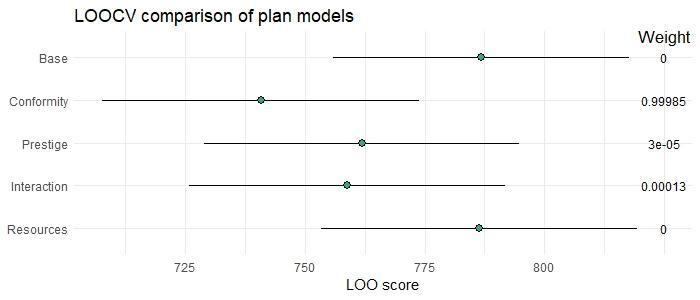 |
| **C** | 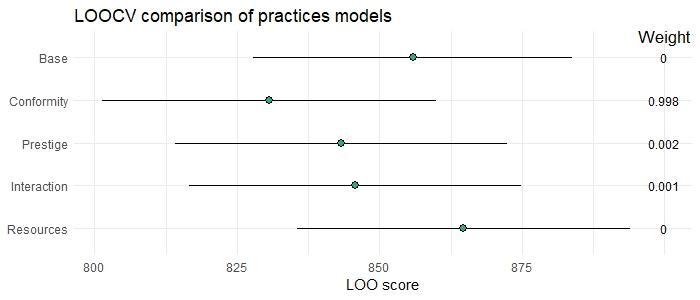 |

Fig S4. PSIS-LOO comparison of the five models for (A) preferences, (B) plans and (C) practices. Green dots and lines mark PSIS-LOO average scores and standard deviations. Lower values indicate less out-of-sample deviance, meaning better performance in predicting new data. Model weights shown on the right indicate that the conformity models score best for preferences, plans and practices.

Table S3. Posterior distributions of the population-level effect sizes, with mean β, 90% credible intervals and the probability that the effect is above zero.

|  | β | CI low | CI high | β (pr > 0) |
| --- | --- | --- | --- | --- |
| Preferences |  |  |  |  |
| Conformity | 2.29 | 1.53 | 3.06 | 99% |
| Prestige | .44 | -0.25 | 1.08 | 87% |
| Years of education | .11 | -.39 | .60 | 64% |
| Rural or urban | .16 | -1.08 | 1.62 | 58% |
| Married | .08 | -.62 | .75 | 57% |
| Emergency money | -.09 | -.81 | .66 | 42% |
| Money | .18 | -.62 | .75 | 64% |
| TLU | .21 | .30 | .76 | 74% |
| Plans |  |  |  |  |
| Perceived majority preference | 2.29 | 1.53 | 3.06 | 99% |
| Prestige | .57 | .09 | 1.07 | 97% |
| Years of education | .18 | -.18 | .54 | 80% |
| Rural or urban | .21 | -.96 | 1.42 | 61% |
| Married | .02 | -.58 | .61 | 53% |
| Emergency money | .17 | -.48 | .87 | 66% |
| Money | .25 | -.44 | .98 | 72% |
| TLU | .02 | .33 | .39 | 54% |
| Practices |  |  |  |  |
| Perceived majority preference | .85 | .00 | 1.69 | 95% |
| Prestige | -.01 | -.50 | .49 | 49% |
| Years of education | .09 | -.27 | .44 | 66% |
| Rural or urban | .43 | -.74 | 1.51 | 75% |
| Visiting Opuwo^1^ | 0.02 | -.25 | .30 | 55% |
| Visiting Hospital^1^ | .35 | -.36 | 1.08 | 79% |
| Married | .53 | -.03 | 1.09 | 94% |
| Emergency money | .09 | -.50 | .67 | 61% |
| Money | .22 | -.40 | .88 | 71% |
| TLU | .03 | .30 | .35 | 55% |


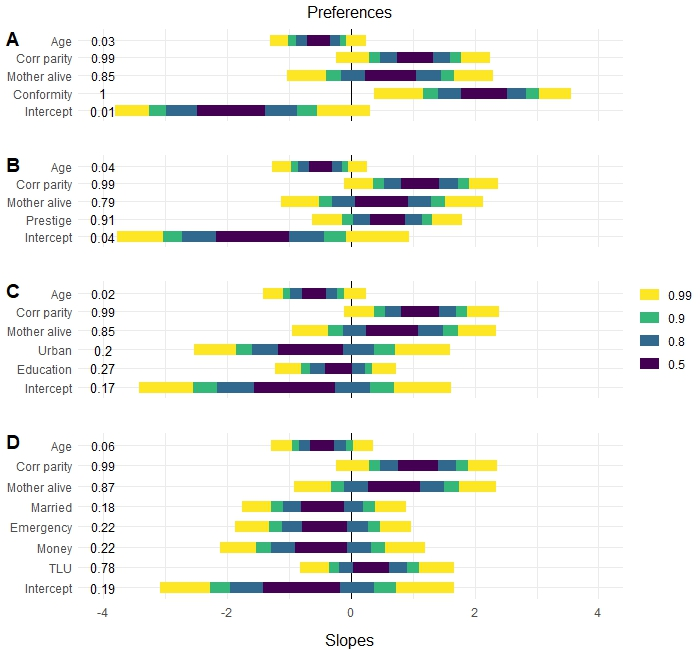


Fig S5. Posterior distributions of the population-level effect sizes, on a subset of potential shifters (individuals who previously practiced the ingroup norm). These effects show the average effects for all norms and villages, as the model included varying intercepts for these variables (shown in S7 and S8). Effect sizes for the models for A) conformity bias, B) prestige bias, C) interaction frequency, and D) access to resources are shown on a logit-scale. The colored bars indicate credible intervals .99 (yellow), .9 (green), .8 (blue) and .5 (dark blue) of the effect sizes. The probability that the effect is positive is shown on the left-hand side of the plots.


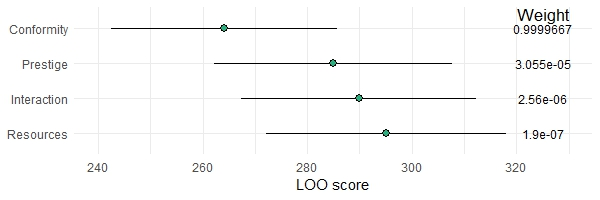


Fig S6. PSIS-LOO comparison of the five models for the preferences of a subset of potential shifters (individuals who previously practiced the ingroup norm). Green dots and lines mark PSIS-LOO average scores and standard deviations. Lower values indicate less out-of-sample deviance, meaning better performance in predicting new data. Model weights shown on the right indicate that the conformity model scores best.

Figure S7. Posterior distribution of the logit-scaled varying intercepts per village in the conformity models on A) preferences, B) plans and C) practices. The colored bars indicate credible intervals .99 (yellow), .9 (green), .8 (blue) and .5 (dark blue) of the effect sizes. The village in the rural area is Omuhanga, the other villages are in the peri-urban area.

| **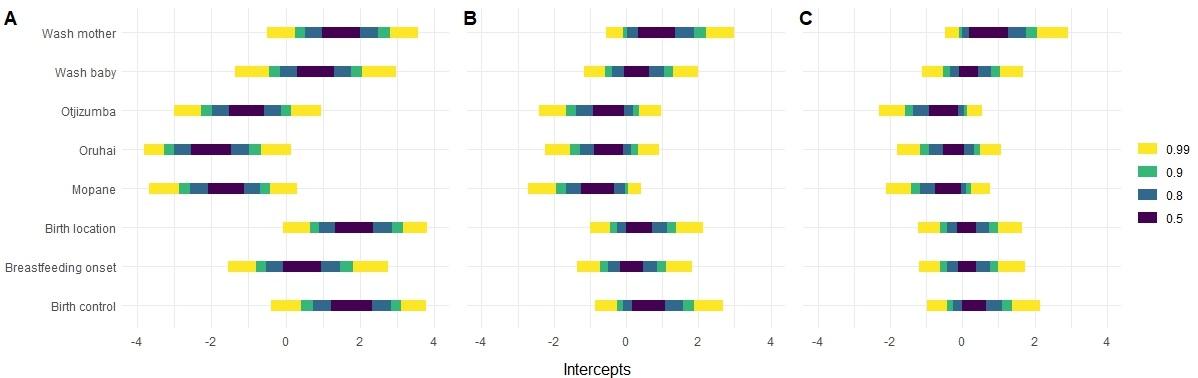** |
| --- |

Figure S8. Posterior distribution of the logit-scaled varying intercepts per norm in the conformity models on A) preferences, B) plans and C) practices. The colored bars indicate credible intervals .99 (yellow), .9 (green), .8 (blue) and .5 (dark blue) of the effect sizes.

## Absolute predictions


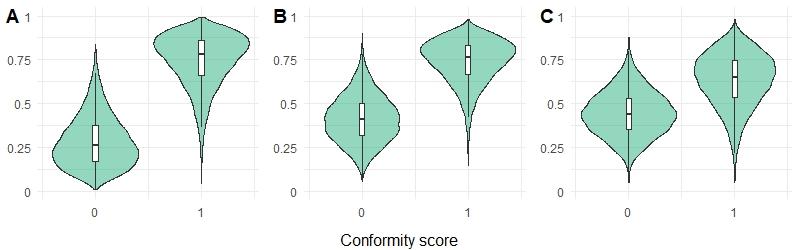


Figure S9. Violin plots of the predicted absolute effects of perceived ingroup majority preference on norm adoption, with the likelihood to adopt a medical recommendation on the x-axis. Predictions are fitted to counterfactual data simulated for a woman of average age and parity (parameter values listed in table S4). Women are consistently more likely to have adopted a medical recommendation in their (A) preferences, (B) plans and (C) practices when they believe most other Himba women prefer that recommendation.

Table S4. The parameter values used in the counterfactual predictions of figures 3, S9 and S10.

| Variable | Value |  |
| --- | --- | --- |
| Conformity | Highest value, lowest value | [0, 1] |
| Age | Mean value | -2.03 |
| Age-corrected parity | Mean value | -3.07 |
| Mother alive | No | 0 |


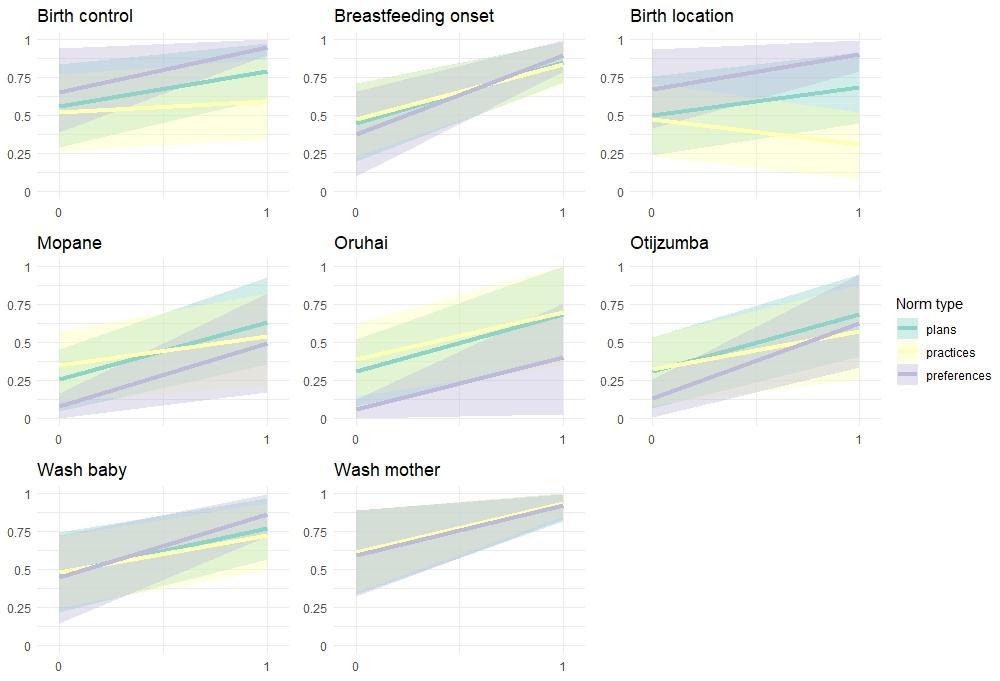


[Fig S10] Predicted absolute effects of perceptions of other women’s norm preferences on one’s own preferences, plans and practices per norm. Predictions are fitted to counterfactual data simulated for a woman of average age and parity (parameter values listed in table S4). Lines and shaded regions indicate the mean and 90% credible interval, with purple for preferences, green for plans, and yellow for practices. Slopes reflect the effect of the variable on norm adoption. In most cases, women who perceive others as preferring a medical recommendation are more likely to adopt that recommendation themselves.
